# Topological disruption of low- and high-order functional networks in presbycusis

**DOI:** 10.1093/braincomms/fcae119

**Published:** 2024-04-06

**Authors:** Yixi Xu, Xiangxiang Li, Qi Yan, Yao Zhang, Song’an Shang, Chunhua Xing, Yuanqing Wu, Bing Guan, Yu-Chen Chen

**Affiliations:** Department of Otolaryngology, Head and Neck Surgery, The Affiliated Lianyungang Hospital of Xuzhou Medical University, Lianyungang 222000, China; Department of Nephrology, Nanjing Yuhua Hospital, Yuhua Branch of Nanjing First Hospital, Nanjing 210006, China; Department of Otolaryngology, Head and Neck Surgery, Clinical Medical College, Yangzhou University, Yangzhou 225001, China; Department of Otolaryngology, Head and Neck Surgery, Clinical Medical College, Yangzhou University, Yangzhou 225001, China; Department of Radiology, Clinical Medical College, Yangzhou University, Yangzhou 225001, China; Department of Radiology, Nanjing First Hospital, Nanjing Medical University, Nanjing 210006, China; Department of Otolaryngology, Nanjing First Hospital, Nanjing Medical University, Nanjing 210006, China; Department of Otolaryngology, Head and Neck Surgery, Clinical Medical College, Yangzhou University, Yangzhou 225001, China; Department of Radiology, Nanjing First Hospital, Nanjing Medical University, Nanjing 210006, China

**Keywords:** presbycusis, functional magnetic resonance imaging, functional connectivity, high-order functional connectivity, graph theory

## Abstract

Prior efforts have manifested that functional connectivity (FC) network disruptions are concerned with cognitive disorder in presbycusis. The present research was designed to investigate the topological reorganization and classification performance of low-order functional connectivity (LOFC) and high-order functional connectivity (HOFC) networks in patients with presbycusis. Resting-state functional magnetic resonance imaging (Rs-fMRI) data were obtained in 60 patients with presbycusis and 50 matched healthy control subjects (HCs). LOFC and HOFC networks were then constructed, and the topological metrics obtained from the constructed networks were compared to evaluate topological differences in global, nodal network metrics, modularity and rich-club organization between patients with presbycusis and HCs. The use of HOFC profiles boosted presbycusis classification accuracy, sensitivity and specificity compared to that using LOFC profiles. The brain networks in both patients with presbycusis and HCs exhibited small-world properties within the given threshold range, and striking differences between groups in topological metrics were discovered in the constructed networks (LOFC and HOFC). NBS analysis identified a subnetwork involving 26 nodes and 23 signally altered internodal connections in patients with presbycusis in comparison to HCs in HOFC networks. This study highlighted the topological differences between LOFC and HOFC networks in patients with presbycusis, suggesting that HOFC profiles may help to further identify brain network abnormalities in presbycusis.

## Teaser

This study highlighted the topological differences between low-order functional connectivity and high-order functional connectivity (HOFC) networks in patients with presbycusis, suggesting that HOFC profiles may help to further identify brain network abnormalities in presbycusis.

## Graphical Abstract

**Graphical Abstract:**
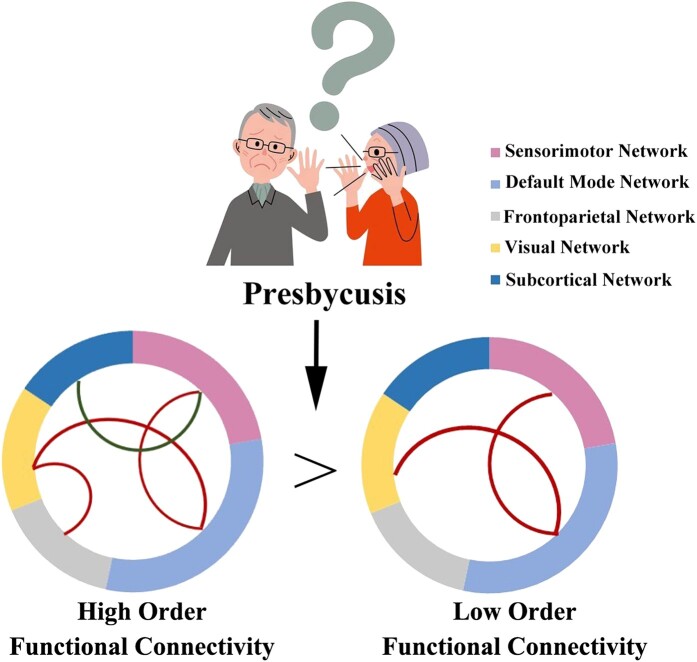

## Introduction

Presbycusis is defined as gradual bilateral high-frequency sensorineural hearing loss and is estimated to affect nearly 35–50% of adults aged 65 years or older.^1^ Presbycusis is characterized by reduced inputs to the primary auditory cortex and receptive language areas of the brain,^2^ seriously affecting verbal communication and semantic understanding,^3^ resulting in serious psychological problems (e.g. social isolation and depression) and becoming a pervasive public health issue. Subsequent studies have inferred that presbycusis is relevant to cognitive decline and added likelihood of developing dementia.^4^ In addition to the applications of hearing aids or artificial cochlea, even after high-quality appropriate therapy, the negative effects of hearing impairment on the patient may continue to last a lifetime.^5^ The investigation of the underlying neuropathological mechanisms of presbycusis is therefore very pivotal.

Postmortem studies of the temporal bones revealed that the major changes in patients with presbycusis are decrease of cochlear hair cells, dysfunction of the stria vascularis and neurodegeneration of the auditory nerve.^6,7^ Recently, with the growing applications of neuroimaging approaches, researchers have found that aberrant spontaneous neural activities in presbycusis occur not only in the auditory cortex but also in the prefrontal cortex and parts of the default mode network (DMN),^8^ indicating that presbycusis is also vulnerable to involvement in other brain regions of the central nervous system. Furthermore, intra- and internetwork functional connectivity (FC) alterations were discovered in patients with presbycusis, and functional network connectivity (FNC) can be applied to forecast latent cognitive decline in the initial stage.^9^ To sum up, resting-state functional magnetic resonance imaging (rs-MRI) is promising for further comprehending the neural mechanism of presbycusis.

Given that FC means the temporal synchronization of neuronal activity between any pair of brain regions,^10^ some studies have used rs-fMRI to construct holistic brain FC networks to investigate presbycusis-related changes in brain activity.^11^ Pearson’s correlation analysis is the most applied FC network construction method due to its simplicity,^12^ and this type of FC network captures the pairwise relationship between regions, which is also known as low-order functional connectivity (LOFC).^13^ Previous graph theory-based studies have uncovered the resultant alterations in network topology in patients with presbycusis, however, these topological abnormalities are rarely detected in patients prior to clinical cognitive impairment.^14^ A new approach referred to as high-order functional connectivity (HOFC) was proposed by Zhang *et al*. captures more complex, higher-level associations among brain regions by measuring the FC topographical similarity of LOFC values between regions one-to-all brain regions, thus, the HOFC indicates comparability of FC topographical profiles and complements the conventional, BOLD-signal synchronization-based LOFC.^15^ Some recent studies indicate that high-level information contains dynamic interaction information among encephalic regions and more nonobjective information of the original data.^16,17^ In this way, HOFC was introduced to capture high-level modulations and has been applied to research of autism spectrum disorder,^18^ early Alzheimer’s disease^19^ and mild cognitive impairment,^20^ yielding the compelling findings related to clinical manifestation and improving the classification performance of the diagnosis.

Previous studies have found that the topological properties are altered in patients with presbycusis in the LOFC networks,^14^ while higher-level interactions were theoretically ignored. Whether HOFC profiles can be used to explore topological alterations in presbycusis has not yet been demonstrated. Given the superiority of HOFC methods, we proposed that HOFC networks could also be used to explore topological anomalies and may serve as effective neuroimaging biomarkers of presbycusis. To demonstrate whether the diagnosis of presbycusis can benefit more from HOFC profiles than LOFC profiles and whether HOFC networks can capture new group difference information and supplement the information captured by the LOFC networks, we first applied LOFC and HOFC profiles to establish brain functional networks of patients with presbycusis and healthy controls (HCs), respectively. Then, we probed the changes in topological properties of the brain functional networks in patients with presbycusis using the graph theory analysis based on the LOFC and HOFC profiles. Considering that presbycusis is not only a destruction of auditory-related networks but also the result of impaired advanced cognitive processes, it is possible that the high-order information interactions in patients with presbycusis could be subtly disclosed in the topological properties of HOFC networks.^14,21^

## Materials and methods

### Participants

A total of 60 patients with presbycusis (26 males/34 females) from the otolaryngology department and 50 matched HCs (24 males/26 females) were enrolled in this study. This study was approved by the Research Ethics Committee of Nanjing Medical University. In order to protect the interests of the subjects, a written informed consent was signed before the experiment began. The hearing threshold was determined by experienced audiologists to measure the pure tone average (PTA) at 0.25, 0.5, 1, 2 and 4 kHz frequencies. The inclusion criteria for the presbycusis group were mean PTA > 25 dB HL in the ear with better hearing and age ≥60 years; for the HC group, PTA ≤ 25 dB HL for all five frequencies was regarded as normal hearing thresholds. In addition, tympanometry was conducted using a middle ear analyzer (GN Otometrics) to validate that the middle ear function was normal. Exclusion criteria for all the participants are shown below: (i) ear diseases that affect hearing threshold, including tinnitus, Meniere’s disease, acoustic neuroma and hyperacusis; (ii) conductive hearing loss > 10 dB in one or both ears (mean difference of air-bone at 0.5, 1, 2 and 4 kHz); (iii) noise exposure history, ototoxic drug therapy, hearing aid use or otologic surgery; (iv) intemperance, brain lesions, stroke and critical illness (e.g. cancer, thyroid dysfunction and anaemia); and (v) MRI contraindications.

### MRI acquisition and data preprocessing

All data were scanned using an MRI scanner (3.0 Tesla, Ingenia, Philips Medical Systems, Netherlands) with an eight-channel phased array head coil under resting conditions. All the participants were instructed to stay awake with their eyes closed and to avoid any deliberate thoughts. Foam pads were used to immobilize their head, and earplugs were used to protect hearing. The rs-fMRI scan went on for 8 and 8 s: repetition time (TR) = 2000 ms, echo time (TE) = 30 ms, slices = 36, thickness = 4 mm, gap = 0 mm, field of view (FOV) = 240 × 240 mm and flip angle (FA) = 90°. The voxel size was 3.75 × 3.75 × 4.0 mm^3^. The structural sequence took 5 and 29 s: TR = 8.1 ms, TE = 3.7 ms, slices = 170, thickness = 1 mm, gap = 0 mm, FA = 8°, acquisition matrix = 256 × 256 and FOV = 256 × 256 mm.

The functional data preprocessing was conducted with GRETNA^22^ (http://www.nitrc.org/projects/gretna/). The preprocessing procedures were indicated below: (i) To alleviate the influence of fluctuations in the image signal at the beginning of the scanning, we dropped the first 5 volumes. (ii) Slice timing calibration, recalibration for head motion correction. Any subjects with displacement >2 mm or rotation >2.0 were excluded from the analysis and no subjects were excluded. (iii) Then, spatial normalization to the EPI template at a resampling of 3 × 3 × 3 mm^3^. (iv) Subsequently, linear detrending and bandpass filtering (0.01–0.08 Hz) were performed for the rs-fMRI time series. (v) At the end of preprocessing, several nuisance signals regression (including signals from white matter, cerebrospinal fluid and the Friston-24 parameters).

### Network construction and analysis

Referring to previous studies, Automated Anatomical Labelling (AAL) brain atlas was applied to divide the brain space into 90 regions of interest (ROIs), and then the mean regional rs-fMRI time series were extracted from each of the 90 brain regions.^23^ We defined each region as one node and the interconnections between regions as the edges of the network. In the subsequent analysis, the obtained mean regional rs-fMRI time series were used to construct LOFC and HOFC networks. We constructed LOFC networks using pairwise Pearson correlation coefficients (PCCs) between regionally averaged rs-fMRI signals for each brain region pair. HOFC networks ware based on the methods proposed by Zhang *et al*.^15^ HOFC networks characterized different pairwise relationships from that of LOFC networks. For LOFC networks, it reflects the comparability of the BOLD time series, whereas HOFC networks measure the comparability of the FC profiles. Before the calculation of the ‘correlation’, all LOFC values were converted to *z*-scores using Fisher’s r-to-z transformation to satisfy the hypothesis of the second round of Pearson’s correlation. After the LOFC and HOFC networks were constructed, features were extracted from the constructed networks using the weighted-graph local clustering coefficients.^16,24^ To lessen the effect of redundant features and enhance the classification performance, the least absolute shrinkage and selection operator (LASSO) was employed to select crucial features for presbycusis disease.^25^ Then, a support vector machine (SVM) was adopted as the ensemble classifier for classification. SVM is an efficient classifier with a simple linear kernel and well-set hyperparameter C, which has superior performance even with a small sample size.^26^ In the subsequent phase, a nested leave-one-out cross-validation (LOOCV) procedure was applied to evaluate the performance of our proposed classification approach.^16^ In addition, to evaluate the classification performance between LOFC and HOFC methods, the area under the receiver operating characteristic curve (AUC), accuracy (ACC), sensitivity (SEN), specificity (SPE) and F-score were calculated.^27^

For network analysis, we employed graph theoretical metrics and network-based statistics (NBS) to characterize topological properties. GRETNA^22^ was applied to calculate global and nodal network metrics, modular architecture, rich-club organization and NBS. The results were visualized by the BrainNet Viewer toolbox.^28^ Global network metrics for the brain networks at each sparsity threshold were calculated for (i) small-world properties, including clustering coefficient (*C*_p_), characteristic path length (*L*_p_), normalized clustering coefficient (Gamma, γ), normalized characteristic path length (Lambda, λ) and small-worldness (Sigma, σ), and (ii) network efficiency parameters, including local efficiency (Eloc) and global efficiency (*E*_glob_). At the nodal level, we explored the betweenness centrality (*B*_c_), degree centrality (*D*_c_) and nodal efficiency (*N*_e_) to assess the centrality of a node. Turning to the modular architecture, the AAL90 template divided the 90 ROIs into five modules (SMN, DMN, FPN, VN and SN).^29^ The mean strength of intra- and intermodular connections was calculated to characterize the basic topological properties of the module, which can be used to estimate modular segregation.^24^ Rich-club nodes were determined by the group-averaged functional network and were defined as the top 10 (12%) brain regions with the highest average nodal degree of all regions in the group;^30^ thus, ‘rich-club’ nodes and ‘peripheral’ nodes were derived. Based on the categorization of the nodes, we classified the edges of the functional network into three types of connections: local connections (linking two peripheral nodes), rich-club connections (linking two rich-club nodes) and feeder connections (linking one rich-club node to one peripheral node).^31^ NBS is often used to detect abnormal subnetworks with altered structural connectivity.^32^ The primary threshold was set to *P* < 0.001 to identify suprathreshold connections; then, the number of edges in subnetworks was determined on the basis of suprathreshold connections. To detect significant subnetworks with a corrected level of *P* < 0.01, 1000 random replicates were completed.

### Statistical analysis

IBM SPSS 21.0 (SPSS, Inc., Chicago, IL, USA) was used to analyse the differences in demographic and clinical characteristics between patients with presbycusis and HCs. The threshold for statistical significance was set to a *P*-value <0.05. The Shapiro–Wilk test was used to assess the normality of the data distribution. Categorical variables were analysed with the chi-squared test (*χ*^2^-test), and normally distributed continuous variables were analysed with a two-sample *t*-test, while nonparametric tests were applied for continuous variables that were identified as not normally distributed. The AUCs of the graph theory parameters were calculated with a sparsity range from 0.06 to 0.40 within an interval of 0.01, and a two-sample *t*-test was used to compare all the network metrics of the structured brain network. For nodal parameters, Bonferroni correction was carried out to correct multiple comparison problems. Two-sample *t*-tests and nonparametric permutation tests with 1000 iterations were used to detect significant intergroup differences in structural connectivity strength on the basis of the NBS method. Age, gender and education were treated as covariates in all the above statistical analyses.

## Results

### Demographics and clinical data

The demographic information and clinical characteristics for all the 110 participants are summarized in Table 1. The presbycusis group and the HC group showed no significant between-group differences in terms of age, gender and education level. PTA in the left and right ears of the presbycusis group was significantly higher than that in the HC group (*P* < 0.001).

**Table 1:** Demographics of patients with presbycusis and HCs

	Presbycusis (*n* = 60)	HCs (*n* = 50)	*P*-value
Age (years)	62.75 ± 7.15	61.08 ± 3.93	0.125
Gender (M/F)	26/34	24/26	0.625
Education levels (years)	10.93 ± 2.02	10.78 ± 1.79	0.677
PTA of left ear (dB HL)	33.10 ± 4.40	17.93 ± 5.84	<0.001^a^
PTA of right ear (dB HL)	33.34 ± 5.96	17.40 ± 5.19	<0.001^a^
Average PTA of both ears (dB HL)	33.22 ± 3.85	17.67 ± 5.12	<0.001^a^

Data are represented as Mean ± SD.

M, male; F, female; PTA, puretone audiometry; HCs, healthy controls.

^a^ *P* < 0.001.

### Classification performance

**Figure 1:**
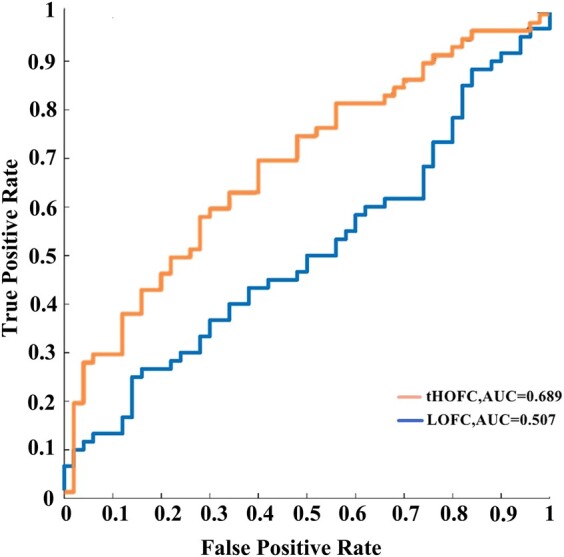
**ROC curve results of LOFC and HOFC networks.** AUC, area under curve; LOFC, low-order functional connectivity; HOFC, high-order functional connectivity.

Table 2 summarizes the comparative classification performance of LOFC and HOFC networks on the basis of functional connectivity characteristics. The classification performance of HOFC networks remarkably outperforms LOFC networks. The use of HOFC profiles boosted patients with presbycusis versus HCs classification accuracy by ∼15.46% (63.64%) and with higher sensitivity (56.67%) and specificity (72.00%) compared to that of LOFC profiles. The ROC curves of the two methods are shown in Fig. 1.

**Table 2:** Classification performance in patients with presbycusis versus HCs differentiation

Method	AUC	ACC (%)	SEN (%)	SPE (%)	*F*-score (%)
LOFC	0.507	48.18	45.00	52.00	48.65
HOFC	0.689	63.64	56.67	72.00	62.96

Abbreviations: AUC, area under the curve; ACC, accuracy; SEN, sensitivity; SPE, specificity; LOFC, low-order functional connectivity; HOFC, high-order functional connectivity.

### Alterations of global topological properties

**Figure 2:**
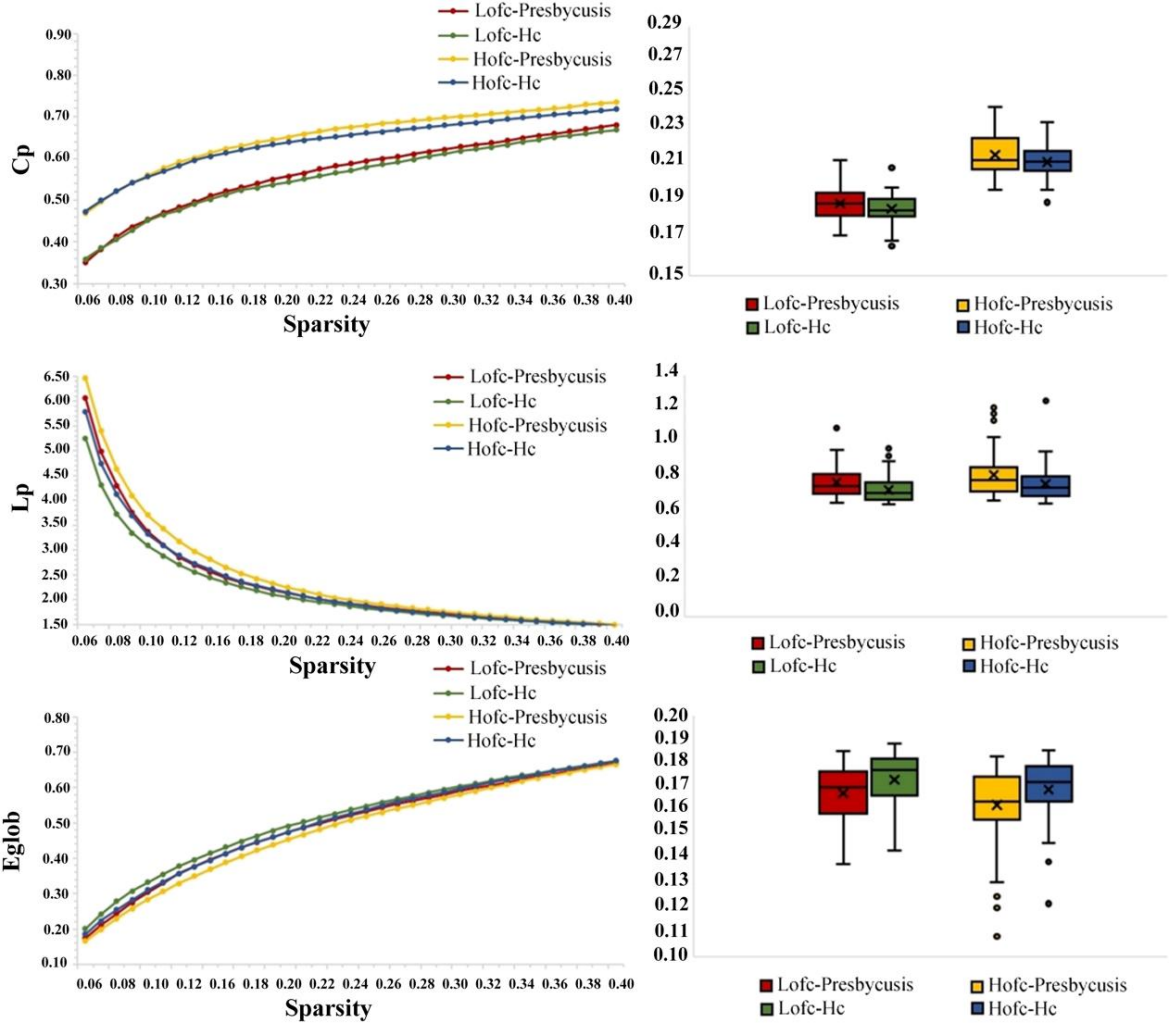
**The differences in global topological properties (*C*_p_, *L*_p_ and *E*_glob_) of LOFC and HOFC networks between presbycusis and HCs**. Two-sample *t*-tests were performed for the global properties between patients with presbycusis and HCs in the LOFC network and HOFC network, respectively. HCs, healthy controls; Cp, clustering coefficient; Lp, characteristic path length; *E*_glob_, global efficiency; LOFC, low-order functional connectivity; HOFC, high-order functional connectivity.

**Figure 3:**
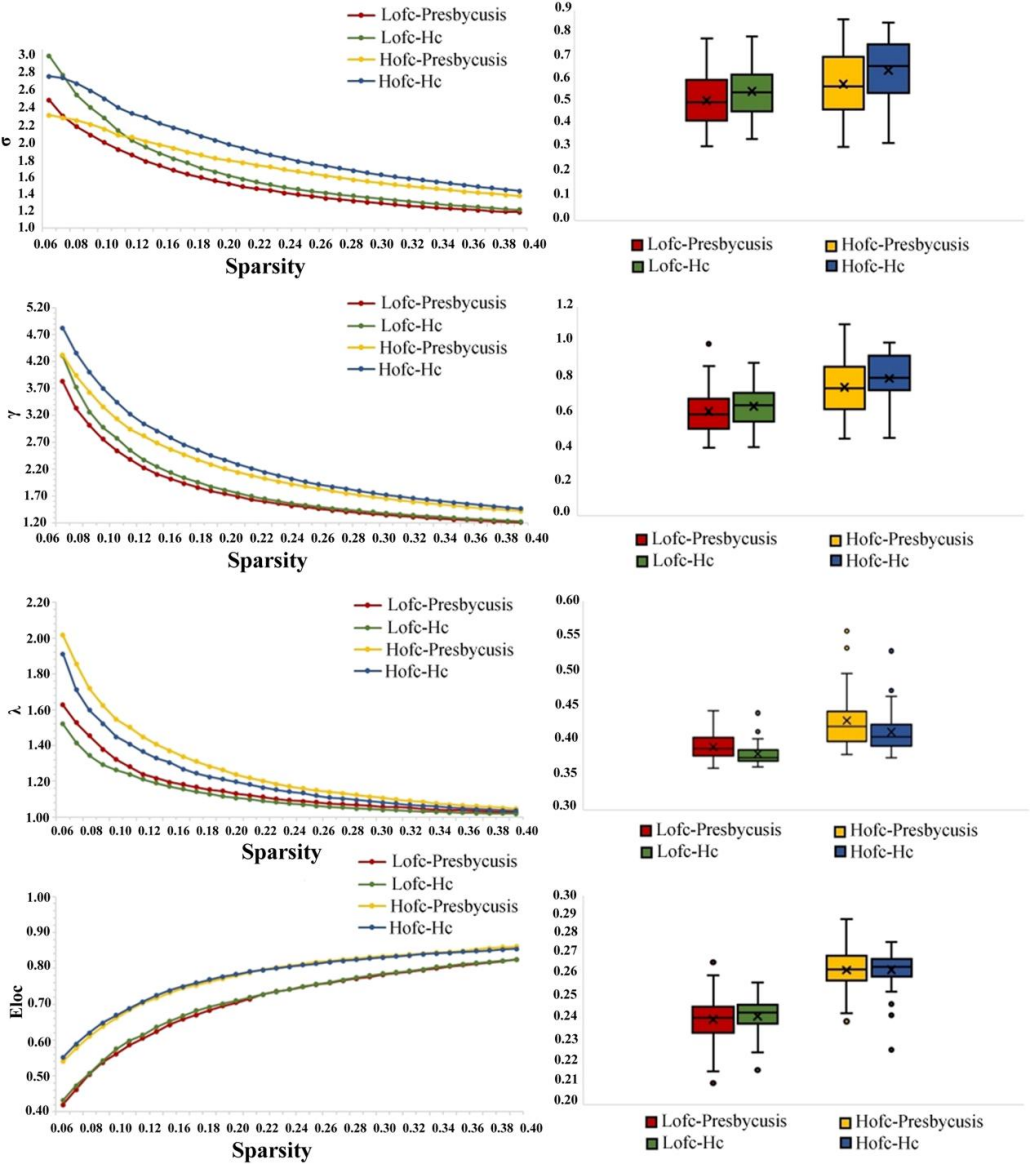
**The differences in global topological properties (Gamma, Lambda, Eloc and Sigma) of LOFC and HOFC networks between presbycusis and HCs.** Two-sample *t*-tests were performed for the global properties between patients with presbycusis and HCs in the LOFC network and HOFC network, respectively. HCs, healthy controls; Gamma, normalized clustering coefficient; Lambda, normalized characteristic path length; Eloc, local efficiency; Sigma, small-worldness; LOFC, low-order functional connectivity; HOFC, high-order functional connectivity.

The topological properties of both groups at the global level are shown in Figs 2 and 3. At the global level, the global indicators generated by both LOFC and HOFC networks show typical small-worldness in the given threshold range (*γ* = *C*_p_/*C*_r_ and > 1, *λ* = *L*_p_/*L*_r_ and ≈ 1, *σ* = *γ*/*λ* > 1). In HOFC networks, the presbycusis group showed significantly increased values in *C*_p_ (*P* = 0.043), *L*_p_ (*P* = 0.023) and *λ* (*P* = 0.015) and decreased values in *E*_glob_ (*P* = 0.018) and *σ* (*P* = 0.023). For LOFC networks, the presbycusis group only showed significantly increased values in *L*_p_ (*P* = 0.009) and *λ* (*P* = 0.005) and significantly decreased values in *E*_glob_ (*P* = 0.015) compared with the HC group.

### Variations in nodal network metrics

**Figure 4:**
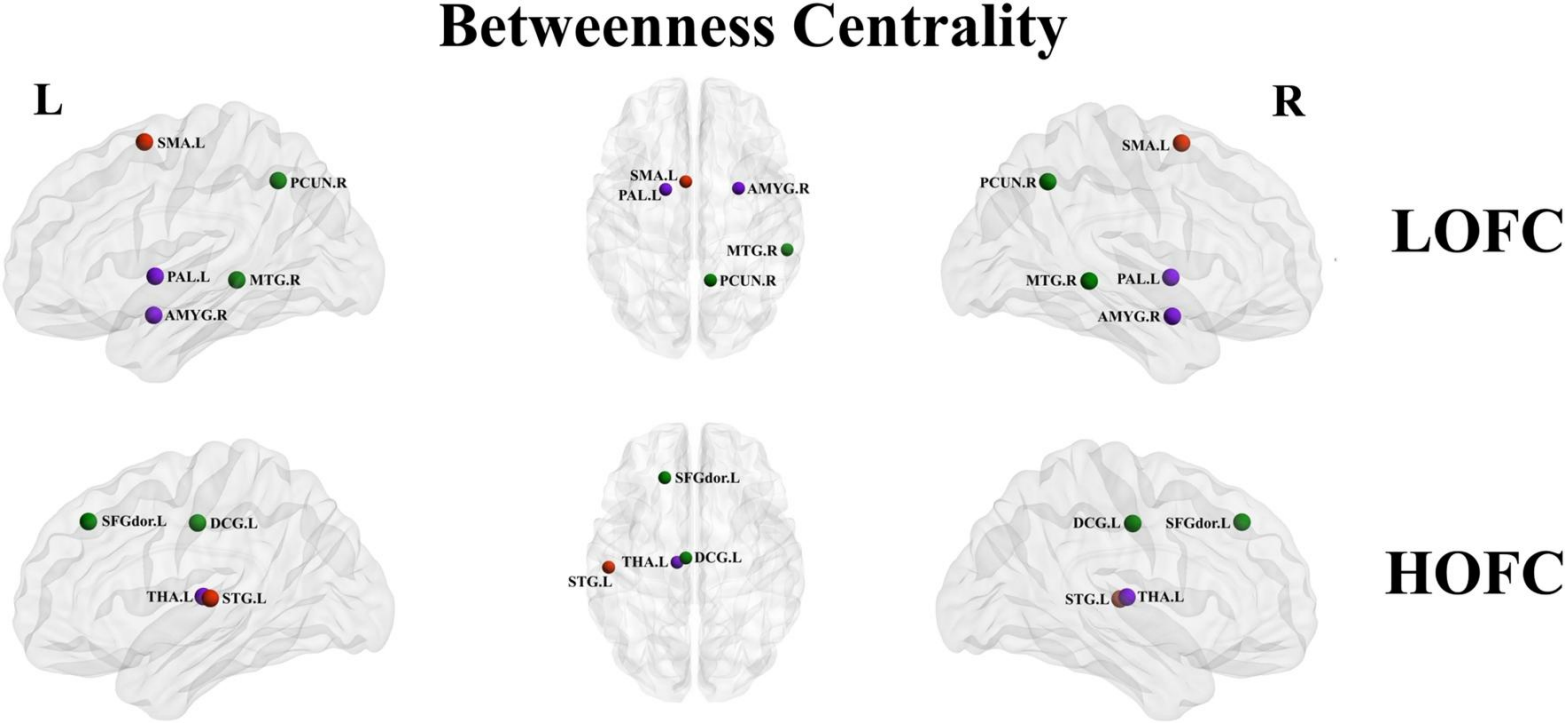
**Nodal betweenness centrality analysis for presbycusis and HCs in LOFC and HOFC networks.** Nodes with significant intergroup differences of nodal centrality groups were indicated with different coloured spheres (*P* < 0.05, Bonferroni corrected, respectively). L, left; R, right; LOFC, low-order functional connectivity; HOFC, high-order functional connectivity; SMA, supplementary motor area; AMYG, amygdala; PCUN, precuneus; PAL, lenticular nucleus, pallidum; MTG, middle temporal gyrus.

Nodes with significant intergroup differences in LOFC and HOFC networks are summarized in Table 3. Based on LOFC profiles, compared with the HC group, the presbycusis group showed increased *B*_c_ in the left Supp_Motor_Area (SMA. L), right amygdala (AMYG. R), right precuneus (PCUN. R), left pallidum (PAL. L) as well as decreased Bc in the right Temporal_Mid (MTG.R). For HOFC networks in the presbycusis group, the Bc of the left Frontal_Sup (SFGdor. L), left Cingulum_Mid (DCG. L), left thalamus (THA. L) decreased compared with the HC group, while the Bc of the left Temporal_Sup (STG. L) increased (Fig. 4).

**Table 3:** Regions showing altered nodal topological metrics in presbycusis group compared with the HCs group of LOFC and HOFC networks

LOFC network			HOFC network		
Brain region	*P*-value		**Brain region**	*P*-value	
**BC**			**BC**		
Supp_Motor_Area_L	0.043	**↑**	Frontal_Sup_L	0.007	**↓**
Amygdala_R	0.044	**↑**	Cingulum_Mid_L	0.046	**↓**
Precuneus_R	0.012	**↑**	Thalamus_L	0.042	**↓**
Pallidum_L	0.032	**↑**	Temporal_Sup_L	0.039	**↑**
Temporal_Mid_R	0.026	**↓**			
**DC**			**DC**		
Frontal_Inf_Oper_R	0.035	**↓**	Frontal_Inf_Oper_R	0.038	**↓**
Frontal_Inf_Tri_L	0.020	**↑**	Frontal_Inf_Tri_L	0.049	**↑**
Supp_Motor_Area_L	0.013	**↑**	Supp_Motor_Area_L	0.017	**↓**
Calcarine_L	0.008	**↑**	Calcarine_L	0.034	**↑**
SupraMarginal_R	0.048	**↓**	SupraMarginal_R	0.008	**↑**
Precuneus_L	0.022	**↑**	Precuneus_L	0.015	**↑**
Precuneus_R	0.009	**↑**	Precuneus_R	0.011	**↑**
			Precentral_R	0.027	**↑**
			Frontal_Sup_L	0.014	**↓**
			Occipital_Sup_L	0.031	**↑**
			Occipital_Sup_R	0.014	**↑**
**NE**			**NE**		
Precentral_R	0.021	**↓**	Precentral_R	0.011	**↓**
Frontal_Inf_Oper_R	0.006	**↓**	Frontal_Inf_Oper_R	0.005	**↓**
Frontal_Inf_Tri_R	0.045	**↓**	Frontal_Inf_Tri_R	0.038	**↓**
Rolandic_Oper_R	0.012	**↓**	Rolandic_Oper_R	0.019	**↓**
SupraMarginal_L	0.026	**↓**	SupraMarginal_L	0.014	**↓**
SupraMarginal_R	0.005	**↓**	SupraMarginal_R	0.002	**↓**
Caudate_L	0.047	**↓**	Caudate_L	0.007	**↓**
Putamen_R	0.033	**↓**	Putamen_R	0.006	**↓**
Temporal_Pole_Sup_R	0.048	**↓**	Temporal_Pole_Sup_R	0.023	**↓**
Temporal_Sup_R	0.043	**↓**	Caudate_R	0.033	**↓**
Occipital_Mid_R	0.043	**↓**	Pallidum_R	0.047	**↓**
Parietal_Inf_R	0.027	**↓**			
Temporal_Mid_L	0.033	**↓**			
Temporal_Mid_R	0.004	**↓**			

Note: Significant *P*-value after Bonferroni correction (*P* < 0.05). The ‘↓’ symbol indicated a significantly lower value (*P* < 0.05) for nodal topological metrics in the presbycusis group compared with the HCs group, while the ‘**↑**’ symbol indicated a significantly higher value (*P* < 0.05) for nodal topological metrics in the presbycusis group compared with the HCs group.

Abbreviations: LOFC, low-order functional connectivity; HOFC, high-order functional connectivity; BC, betweenness centrality; DC, degree centrality; NE, nodal efficiency.

**Figure 5:**
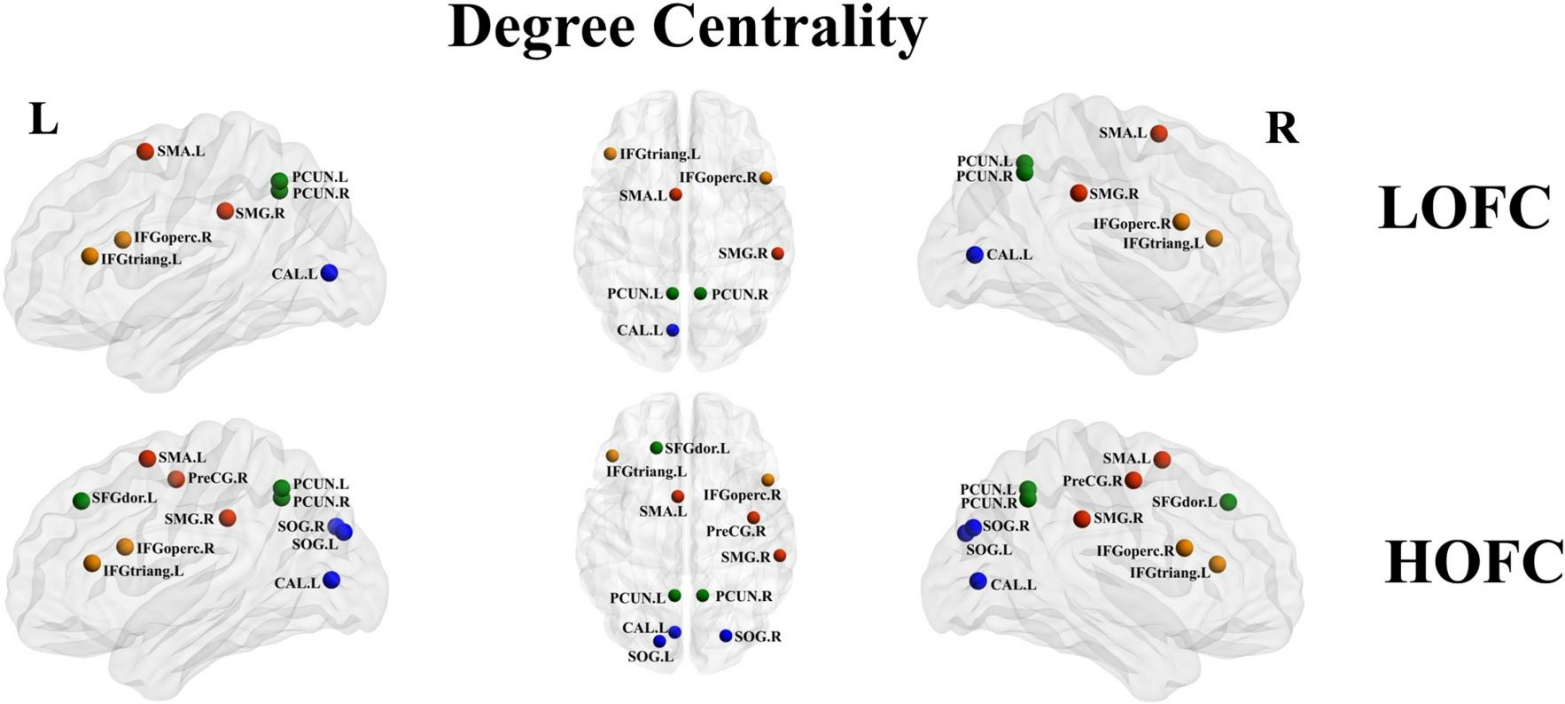
**Nodal degree centrality analysis for presbycusis and HCs in LOFC and HOFC networks.** Nodes with significant intergroup differences of nodal centrality groups were indicated with different coloured spheres (*P* < 0.05, Bonferroni corrected, respectively). L, left; R, right; LOFC, low-order functional connectivity; HOFC, high-order functional connectivity; SMA, supplementary motor area; PCUN, precuneus; IFGtriang, inferior frontal gyrus, triangular part; CAL, calcarine fissure and surrounding cortex; IFGoperc, inferior frontal gyrus, opercular part; SFGdor, superior frontal gyrus, dorsolateral; SMG, supramarginal gyrus; SOG, superior occipital gyrus; PreCG, precental gyrus.

Compared with the HC group, the LOFC network-based Dc of the presbycusis group was significantly increased in the left Frontal_Inf_Tri (IFGtriang. L), left Supp_Motor_Area (SMA. L), left calcarine (CAL. L), bilateral precuneus (PCUN. L and PCUN. R) as well as decreased Dc in the right Frontal_Inf_Oper (IFGoperc. R) and right supramarginal gyrus (SMG.R). In HOFC networks, compared with the HC group, in addition to the above changes contained in the LOFC networks, the presbycusis group showed significantly higher Dc in the left Frontal_Sup (SFGdor. L), bilateral Occipital_Sup (SOG. L and SOG. R) and lower Dc in the right precentral (PreCG. R) (Fig. 5).

**Figure 6:**
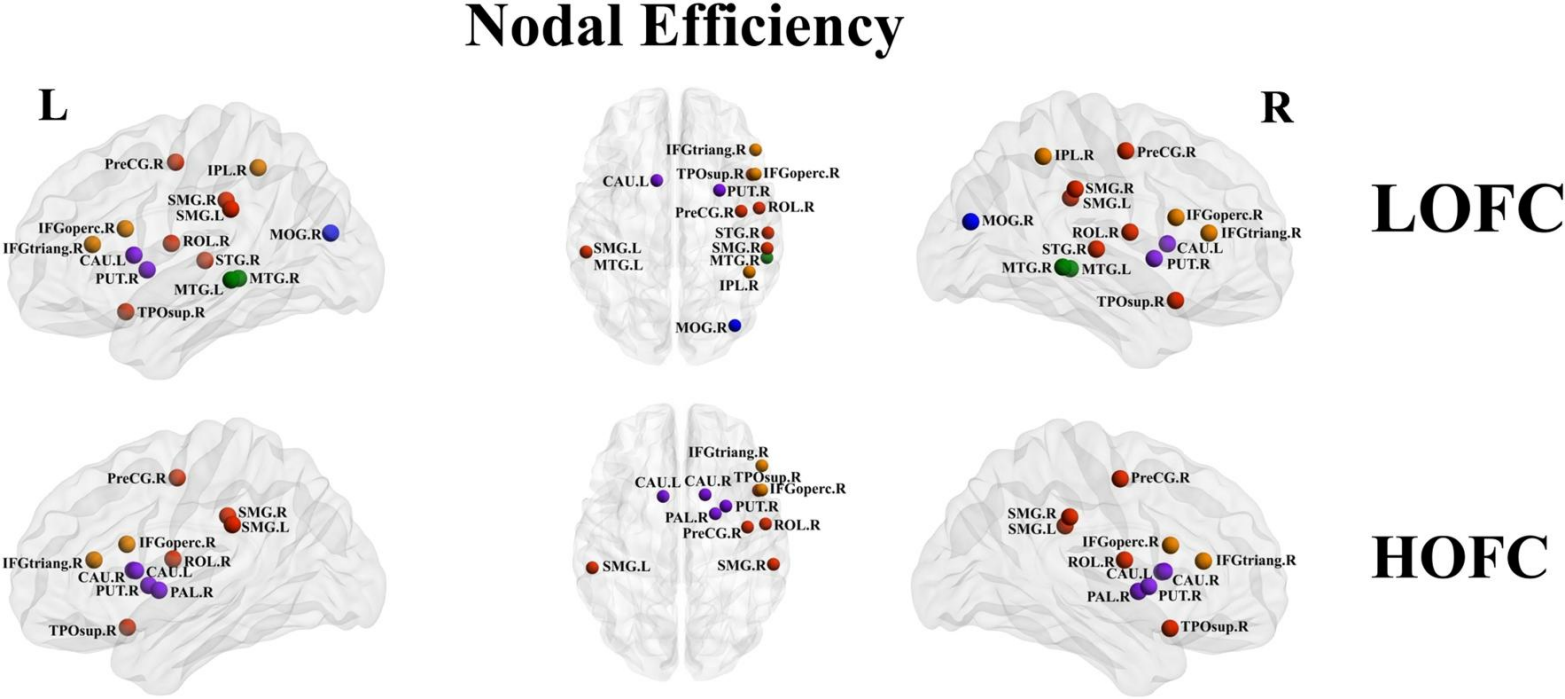
**Nodal efficiency analysis for presbycusis and HCs in LOFC and HOFC networks.** Nodes with significant intergroup differences of nodal centrality groups were indicated with different coloured spheres (*P* < 0.05, Bonferroni corrected, respectively). L, left; R, right; LOFC, low-order functional connectivity; HOFC, high-order functional connectivity; PAL, lenticular nucleus, pallidum; MTG.L, middle temporal gyrus; STG, superior temporal gyrus; IFGtriang, inferior frontal gyrus, triangular part; IFGoperc, inferior frontal gyrus, opercular part; SMG, supramarginal gyrus; SOG, superior occipital gyrus; PreCG, precental gyrus; ROL, rolandic operculum; MOG, middle occipital gyrus; IPL, inferior parietal lobule; CAU, caudate nucleus; PUT, lenticular nucleus, putamen; TPOsup, temporal pole: superior temporal gyrus.

With LOFC profiles, compared with the HC group, the presbycusis group showed decreased Ne in the right precentral (PreCG. R), right Frontal_Inf_Ope (IFGoperc. R), right Frontal_Inf_Tri (IFGtriang. R), right Rolandic_Oper (ROL. R), right Occipital_Mid (MOG. R), right Parietal_Inf (IPL. R), bilateral supramarginal (SMG. L and SMG. R), left Caudate (CAU. L), right putamen (PUT. R), right Temporal_Sup (STG. R), right Temporal_Pole_Sup (TPOsup. R) and bilateral Temporal_Mid (MTG. L and MTG.R). In HOFC networks, compared with the HC group, the presbycusis group showed decreased Ne in the right precentral (PreCG. R), right Frontal_Inf_Ope (IFGoperc. R), right Frontal_Inf_Tri (IFGtriang. R), right Rolandic_Oper (ROL. R), bilateral supramarginal (SMG. L and SMG. R), bilateral Caudate (CAU. L and CAU. R), right putamen (PUT. R), right pallidum (PAL. R) and right Temporal_Pole_Sup (TPOsup. R) (Fig. 6).

### Aberrations of modular architecture

**Figure 7:**
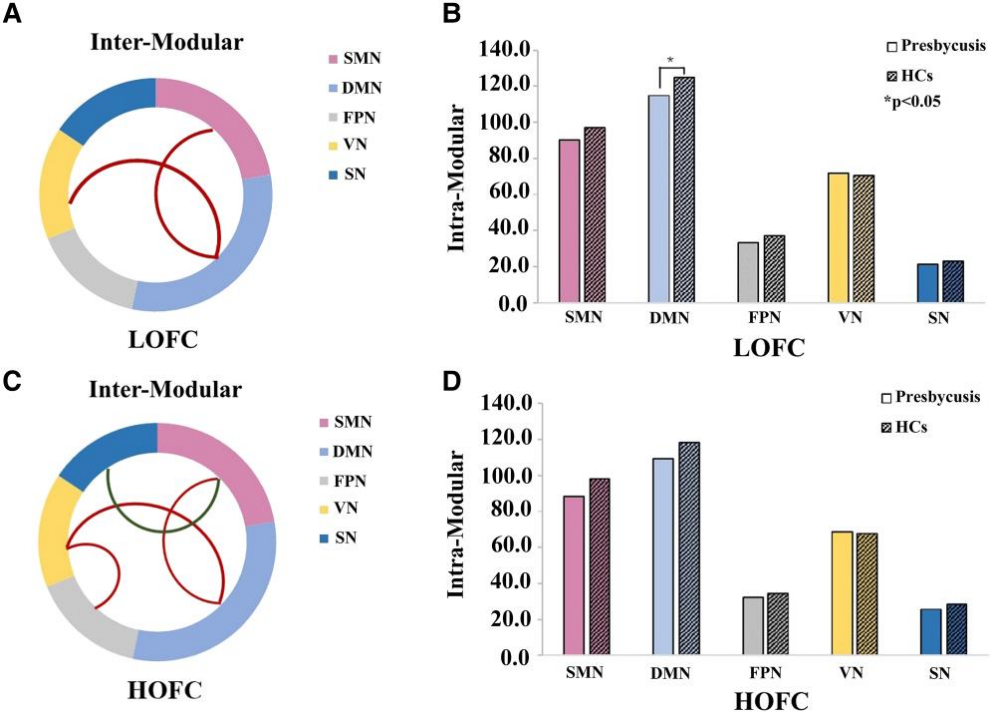
**Aberrant modular architectures of LOFC and HOFC networks for each group.** Significantly altered inter-modular functional connectivity between groups was observed in LOFC (**A**) and HOFC (**C**) networks (*P* < 0.05, respectively). Intra-modular functional connectivity between groups was compared using a two-sample *t*-test in LOFC (**B**) and HOFC networks (**D**), and significant changes were observed (*P* < 0.05, respectively). * indicates significant intergroup difference with *P* < 0.05. LOFC, low-order functional connectivity; HOFC, high-order functional connectivity; HCs, healthy controls; SMN, sensorimotor network; DMN, default mode network; FPN, fronto-parietal network; VN, visual network; SN, subcortical network.

For LOFC networks in the presbycusis group, the intermodular connective strengths showed a significant increase between the SMN and DMN (*P* = 0.006) and between the DMN and VN (*P* = 0.008) compared with the HC group (Fig. 7A), while intramodular connective strengths showed a significant decrease within the DMN (*P* = 0.048) (Fig. 7B). For HOFC networks in the presbycusis group, the intermodular connective strengths showed a significant increase between the SMN and DMN (*P* = 0.002), the DMN and VN (*P* = 0.010) and the FPN and VN (*P* = 0.026) and a significant decrease between the SMN and SN (*P* = 0.032) compared with the HC group (Fig. 7C). However, there were no significant differences in intramodular connective strengths between groups in HOFC networks (Fig. 7D).

### Rich-club organization and reorganization

**Figure 8:**
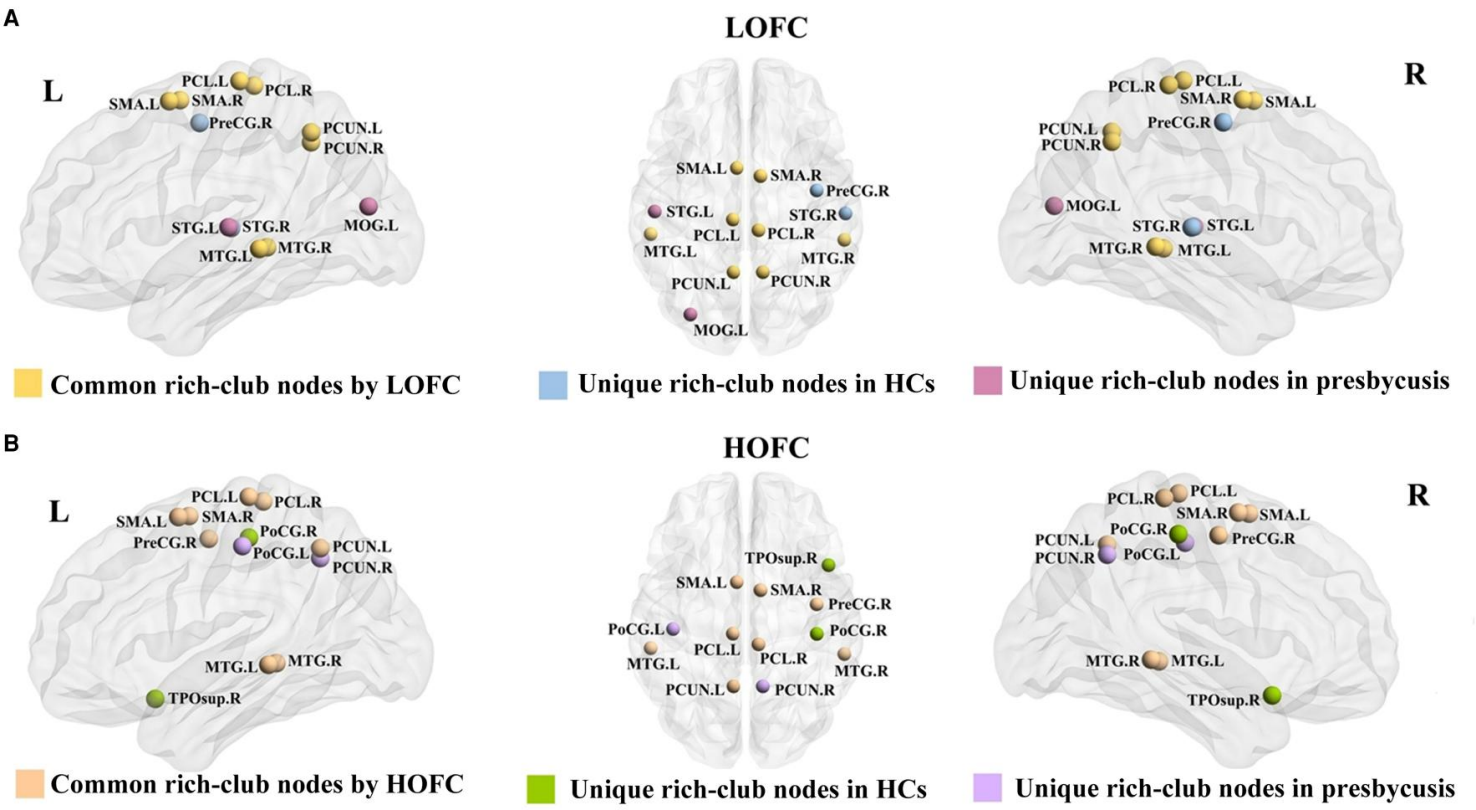
**The distribution of rich-club nodes in the whole brain for each group in LOFC and HOFC networks.** (**A**) Common and unique rich-club nodes shared by patients with presbycusis and HCs in LOFC networks. (**B**) Common and unique rich clubs nodes shared by patients with presbycusis and HCs in HOFC netwroks. LOFC, low-order functional connectivity; HOFC, high-order functional connectivity; HCs, healthy controls; SMA, supplementary motor area; PCUN, precuneus; PCL, paracentral lobule; MTG, middle temporal gyrus; MOG, middle occipital gyrus; STG, superior temporal gyrus; PreCG, precental gyrus; PoCG, postcentral gyrus; TPOsup, temporal pole: superior temporal gyrus.

**Figure 9:**
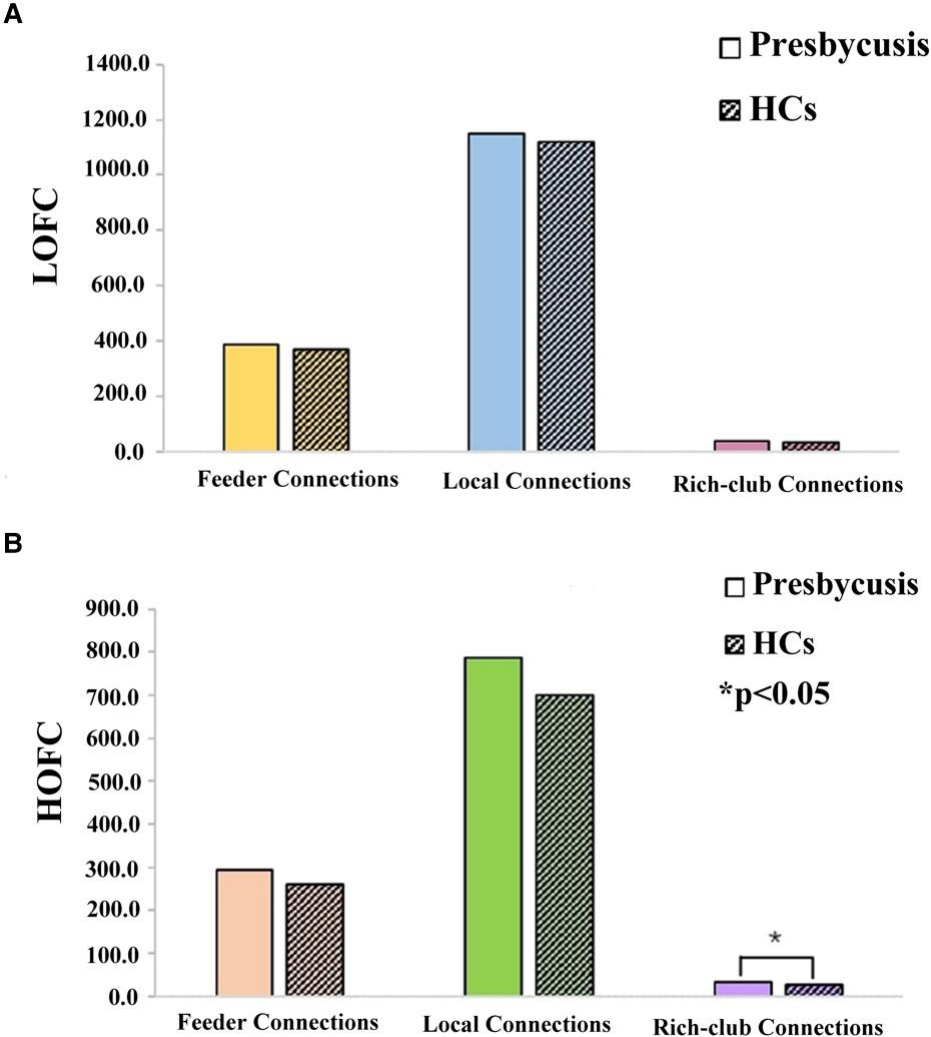
**Comparison of the three types of connections for each group in LOFC and HOFC networks.** The mean strength of the three connections between patients with presbycusis and HCs were compared using a two-sample *t*-test. In LOFC networks, no significant inter-group differences were observed in the mean strength of the three connections (**A**). While in HOFC networks, the mean strength of rich-club connections in patients with presbycusis was significantly higher than that in HCs (*P* < 0.05) (**B**). *indicates significant intergroup difference with *P* < 0.05.

Based on LOFC profiles, the rich-club nodes in the presbycusis group were mainly distributed in the bilateral Supp_Motor_Area (SMA. L and SMA. R), bilateral precuneus (PCUN. L and PCUN. R), bilateral paracentral lobule (PCL. L and PCL. R), bilateral Temporal_Mid (MTG. L and MTG. R), left Occipital_Mid (MOG. L) and left Temporal_Sup (STG. L) and the rich-club nodes in the HC group were mainly distributed in the bilateral Supp_Motor_Area (SMA. L and SMA. R), bilateral precuneus (PCUN. L and PCUN. R), bilateral paracentral lobule (PCL. L and PCL. R), bilateral Temporal_Mid (MTG. L and MTG. R), right precentral (PreCG. R) and right Temporal_Sup (STG. R) (Fig. 8A). However, on the basis of HOFC networks, the newly formed rich-club nodes in the presbycusis group were mainly distributed in the bilateral Supp_Motor_Area (SMA. L and SMA. R), bilateral precuneus (PCUN. L and PCUN. R), bilateral paracentral lobule (PCL. L and PCL. R), bilateral Temporal_Mid (MTG. L and MTG. R), right precentral (PreCG. R) and left postcentral (PoCG. L) (Fig. 8B). In LOFC networks, no significant group differences were observed in the mean strength of the three connections between the presbycusis group and the HC group (Fig. 9A). In HOFC networks, compared with the HC group, the presbycusis group showed a significant increase in rich-club connections (*P* = 0.046) (Fig. 9B).

### Network-based statistics

**Figure 10:**
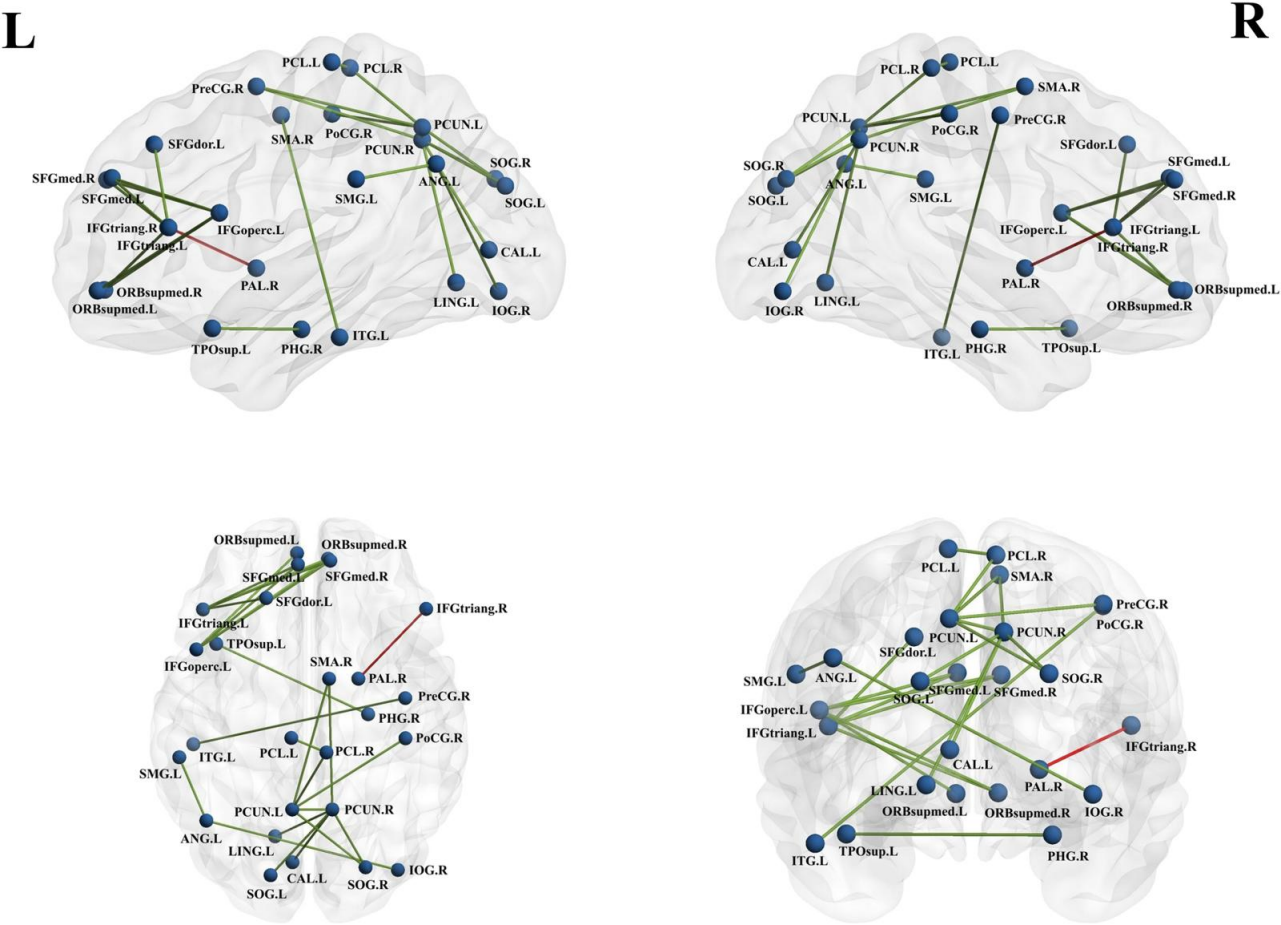
**NBS analysis revealed significant altered functional connections featured highly connected nodes of presbycusis in HOFC networks (*p* < 0.05, NBS corrected).** Spheres represent regions (node) and lines indicate connections (edge). L, left; R, right; PreCG, precental gyrus; SFGdor, superior frontal gyrus, dorsolateral; IFGoperc, inferior frontal gyrus, opercular part; IFGtriang, inferior frontal gyrus, triangular part; SMA, supplementary motor area; SFGmed, superior frontal gyrus, medial; ORBsupmed, superior frontal gyrus, medial orbital; PHG, parahippocampal gyrus; CAL, calcarine fissure and surrounding cortex; LING, lingual gyrus; SOG, superior occipital gyrus; IOG, inferior occipital gyrus; PoCG, postcentral gyrus; SMG, supramarginal gyrus; ANG, angular gyrus; PCUN, precuneus; PCL, paracentral lobule; PAL, lenticular nucleus, pallidum; TPOsup, temporal pole: superior temporal gyrus; ITG, inferior temporal gyrus.

In HOFC networks, we used NBS analysis to identify a subnetwork containing 26 nodes and 23 significantly altered internodal connections that were significantly different between groups (*P* < 0.05, NBS corrected) (Fig. 10). The nodes were mainly located in the DMN regions, SMN regions, VN regions, FPN regions and SN regions. The connections involved linking between different regions, including decreased FC in the DMN and FPN regions, the DMN and SMN regions, the DMN and VN regions, the SMN and SN regions and increased FC in the SN and FPN regions. In addition, there were two decreased connections within the regions, including decreased FC within the DMN regions and the SMN regions. However, no subnetwork of altered structural connectivity was found in patients with presbycusis in LOFC methods.

## Discussion

In the present study, HOFC networks were applied to characterize the topology of high-level brain functional networks of patients with presbycusis. Our study had three major findings: (i) Small-world topological organization was exhibited in both the presbycusis group and the HC group in both LOFC and HOFC networks. Relative to the matched HC group, high-level information interactions in the presbycusis patients were disrupted and showed prominent changes in whole-brain topological metrics. (ii) HOFC profiles achieve greater sensitivity and accuracy than LOFC profiles in the detection of in the detection of network disruptions prior to clinical evidence of cognitive impairment in presbycusis. (iii) As a new method for measuring brain functional organization, HOFC profiles can provide supplementary information for traditional FC-based networks and obtain higher-level information interactions of human brain, so as to better understand the functional organization of our brain and the disturbances caused by its diseases.

Traditional FC-based networks, i.e. LOFC networks, rely on BOLD signal temporal synchronization, and this one-to-one pairwise FC calculation uncovers only simple temporal synchronization between two brain regions. On this basis, one-to-all topographical FC profiles were used as high-level features of a certain ROI, and HOFC networks were then calculated with a second level of correlation on the features between each pair of ROIs.^15^ Previous studies have mainly concentrated on the exploration of the LOFC-based topological reorganization of the presbycusis;^11,33^ however, presbycusis might affect not only the relationship between two regions but also the higher-level relationship among multiple brain regions. To better understand the abnormalities in brain topological networks caused by presbycusis, HOFC networks were applied to capture complex interactions among brain regions. HOFC methods achieved a better classification effect compared with LOFC methods, which can improve the classification accuracy and sensitivity by 15.5% and 11.7% on average. It follows that HOFC methods can capture high-order characteristics of the brain network to enhance the classification of diseases and have been demonstrated in studies of type 2 diabetes with or without cognitive disorder^25^ and mild cognitive impairment.^15^ Hence, we proposed that HOFC networks may serve as a practicable approach to study presbycusis and have broad prospects in clinical applications.

We contrasted the graph theory properties of the complicated brain network extracted by LOFC and HOFC methods between the presbycusis group and the HC group. Small-world architecture has a high clustering coefficient and short characteristic path length, which is conducive to local specialized processing and global distributed processing,^34^ striking a balance between integration and segregation to realize fast and efficient information processing.^35^ Both LOFC and HOFC methods shared the common changes in *L*_p_, *λ* and *E*_glob_ of the presbycusis group, which were commonly used to evaluate the integration ability of the whole-brain, and the disrupted functional integration (increased *L*_p_, *λ* and decreased *E*_glob_) in the presbycusis group implied a limited ability of global information communication.^36^ Profiting from the sensitivity of HOFC to high-level functional interactions,^13^ this study exposed that the HOFC networks in HC group exhibit typical small-world properties, suggesting that high-level functional interactions still maintain an effective network to equilibrium local specialization and global integration. In addition to the topological reorganization of brain functional networks in presbycusis reflected by LOFC methods, HOFC methods also reflected the changes in other graph theory indicators. *C*_p_ is usually used to assess segregation ability,^37^ and the improved functional segregation (increased *C*_p_) in presbycusis patients suggested that the separation of information flow for region-specific processing is more efficient. The decreased sigma indicated that the disequilibrium in the differentiation and integration of brain networks existed in patients with presbycusis.^14^ HOFC-based graph theory analysis not only revealed substantially new information but also provided supplementary information to LOFC networks. Given the frequent occurrence of cognitive decline in patients with long-term hearing loss, it can be speculated that there exists a correlation between impairment functional networks at high levels and neurodegeneration related to cognition prior to clinical manifestation.

Markedly different nodal parameters, including Bc, Dc and Ne, were found in presbycusis in both LOFC and HOFC networks, indicating nodal reorganization of the whole-brain functional network. Bc measures the influence of a node on the overall flow of information in the graph, Dc evaluates the importance of a node in the network, and Ne represents the information communication capability of a node.^24,38^ Alterations in nodal topological properties can alter the connectivity and communication efficiency with other regions, resulting in abnormalities in regional neural circuits of the functional brain network, which can provide additional information not available from the investigation of the global topological network.^38^ The nodal metrics comparison results for LOFC and HOFC did not simply overlap, HOFC-based analysis brought new findings that were complementary to LOFC. The temporal lobe is mainly in charge of auditory signals^39^ and the caudate nucleus is thought to be associated with executive dysfunction in Parkinson’s patients.^40^ The precuneus is thought to be involved in motor, visual and cognitive functions,^41^ and studies^14,36^ have proven that the precuneus can serve as a compensatory node. The compensatory plasticity of these nodes enables them to maintain effective information exchange through the functional network of patients with presbycusis.

A module is a group of nodes in the brain network that are firmly connected within a local area but loosely connected externally.^42^ Functional segregation of the brain refers to the ability for specialized processing in densely interconnected groups of brain regions and the modular architecture can estimate the altered segregation more accurately than the global topological properties.^24^ When comparing the two groups, HOFC networks exhibited a more prominent modular architecture than LOFC networks. The FPN can be considered as a language network and the SN is mainly connected with social cognition and executive function.^43^ Thus, the hyperconnectivity between modules and the hypoconnectivity within modules may explain the decline in verbal communication and auditory perception in patients with long-term hearing loss.^44^ Therefore, HOFC profiles are able to compensate for our traditional understanding of brain connectome, which is mainly based on LOFC networks analysis. Furthermore, the distribution pattern of rich-club nodes in patients with presbycusis was different from that in HCs, indicating that the architecture of rich-club nodes was reorganized in presbycusis. By separately using LOFC and HOFC methods, we discovered that the newly formed rich-club nodes of presbycusis were distributed in different modules. Previous studies have suggested that the concatenation among rich-club nodes is essential for the integration of information among different subsystems of the human brain.^31^ Given that the increased rich-club connections in HOFC networks, we proposed that HOFC networks can reveal the high-level functional reorganization to make up for the dysfunction of the node.

In the HOFC networks-based NBS, decreased structural connections within the DMN were observed in the presbycusis group in the present study. The DMN, a network associated with self-referential activity in the brain, is active at rest and suppressed during tasks.^45^ In addition, it has been linked to physiological functions such as cognition and emotion.^46^ Previous studies have linked disrupted DMN connectivity to cognitive decline in patients with type 2 diabetes.^47^ Thus, disrupted connectivity in DMN might reflect cognitive deficits in presbycusis patients, which is also consistent with the clinical manifestations of cognitive decline in presbycusis patients with long-term hearing loss. In addition, decreased internodal connections between DMN–FPN, DMN–VN and DMN–SMN were detected, suggesting their decreased roles in integrating information in brain networks. FPN is regarded as a language network,^48^ VN and SMN play important roles in visual information processing^49^ and sensorimotor function.^50^ Hearing loss is thought to lead to reduced central auditory activation, which negatively affects auditory perception and verbal communication ability.^44^ This study supports that the decreased connections among these brain regions may be associated with dysfunction of sensory, cognitive and speech function in patients with presbycusis. What’s more, we found that patients with presbycusis showed remarkably increased structural connections between SN and FPN. This could be explained by auditory cortical plasticity and functional compensation, suggesting that presbycusis patients tend to solicit more resources to support auditory perception, similar to those of previous studies.^44^ Given that presbycusis is associated with impaired high-level cognitive function and increased risk of dementia,^51^ it is plausible that the high-level information interaction dysfunction in presbycusis could be sensitively revealed in the topological properties of HOFC networks.

Several limitations of the current study should be recognized. First, the statistical power of our study needs further validation in a larger cohort in the future. Second, although differences in topological properties in presbycusis between LOFC and HOFC networks were found by using the AAL90 template, the use of multiple templates in subsequent studies will help further validate the value of HOFC networks. Third, as a preliminary study, this study aims to compare HOFC with traditional LOFC, so dynamic FNC is not considered. However, studies based on dynamic FNC will be of great significance in exploring the abnormalities of higher-order brain functional activities in presbycusis.

## Conclusions

Our study indicates that HOFC methods can reflect the injurious changes of higher-order topological network properties in patients with presbycusis. Although both LOFC and HOFC networks can serve as effective neurologic markers for detecting presbycusis, HOFC network achieves better sensitivity and accuracy than LOFC network in the detection of presbycusis-induced brain functional changes. As an important complement to LOFC methods, HOFC methods are helpful to further investigate the brain network abnormalities of neurological disease and deepen the understanding of the central mechanism of disease.

## Data Availability

The data that support the findings of this study are available on request from the corresponding author. The data are not publicly available due to privacy or ethical restrictions.
